# Transdiaphragmatic Intercostal Herniation following Blunt Trauma

**DOI:** 10.1155/2012/502765

**Published:** 2012-11-01

**Authors:** Debkumar Sarkar, Melissa Warta, Jason Solomon

**Affiliations:** Department of Radiology, Cooper University Hospital, Cooper Medical School of Rowan University, One Cooper Plaza, B23, Camden, NJ 08103, USA

## Abstract

Intercostal herniation is very rarely and sporadically reported in the literature. Intercostal hernia can occur following blunt trauma and may be associated with rib fractures. We present a case of a patient who presented with rib fractures, diaphragmatic rupture, and intrathoracic herniation of abdominal contents with subsequent herniation of both lung and abdominal contents through an intercostal defect. The patient was successfully treated with primary surgical repair of the diaphragm and intercostal hernia. The presentation, pathophysiology, and management of this rare clinical entity are discussed.

## 1. Case Report

A 59-year female presented via helipad as a trauma alert following head on motor vehicle collision with a tractor trailer. The patient was a restrained driver and denied loss of consciousness or head trauma. On arrival, she was awake, alert, and complaining only of right ankle pain. Her past medical history included hypertension, schizophrenia, anxiety, uterine cancer, and obesity. She had a prior surgical history of bilateral total hip arthroplasty. On physical examination the patient had multiple contusions and abrasions including an abdominal seat belt sign with several areas of ecchymosis and skin abrasions across the lower abdomen as well as a seatbelt contusion across the anterior chest wall.

**Figure 1:**
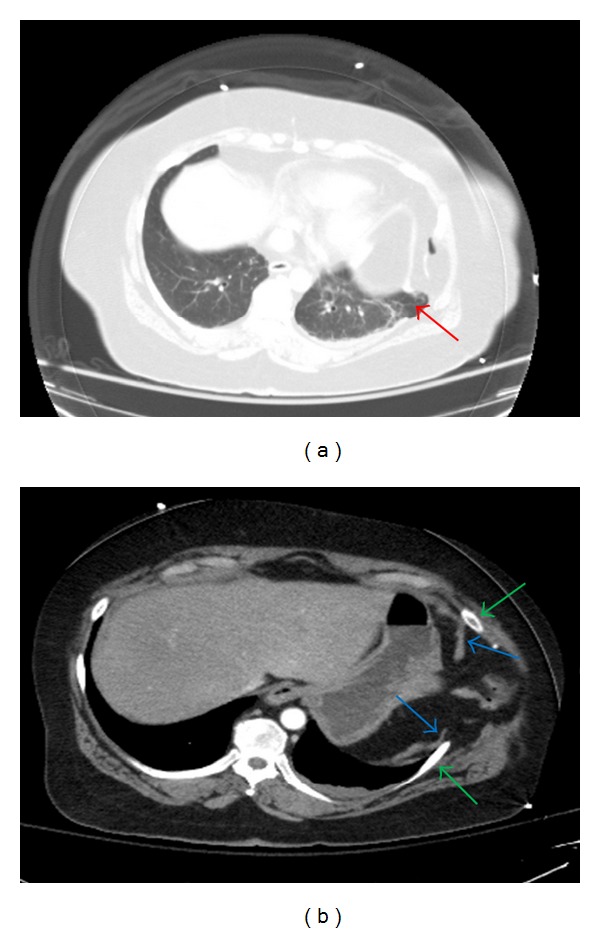
Axial CT images of the chest demonstrate herniation of lung beyond the thoracic cavity (red arrow) (a). There is discontinuity of the diaphragm (blue arrows) and widened intercostal space (green arrows). Herniation of abdominal contents is present including omentum and transverse colon into the chest and through the intercostal defect (b).

**Figure 2:**
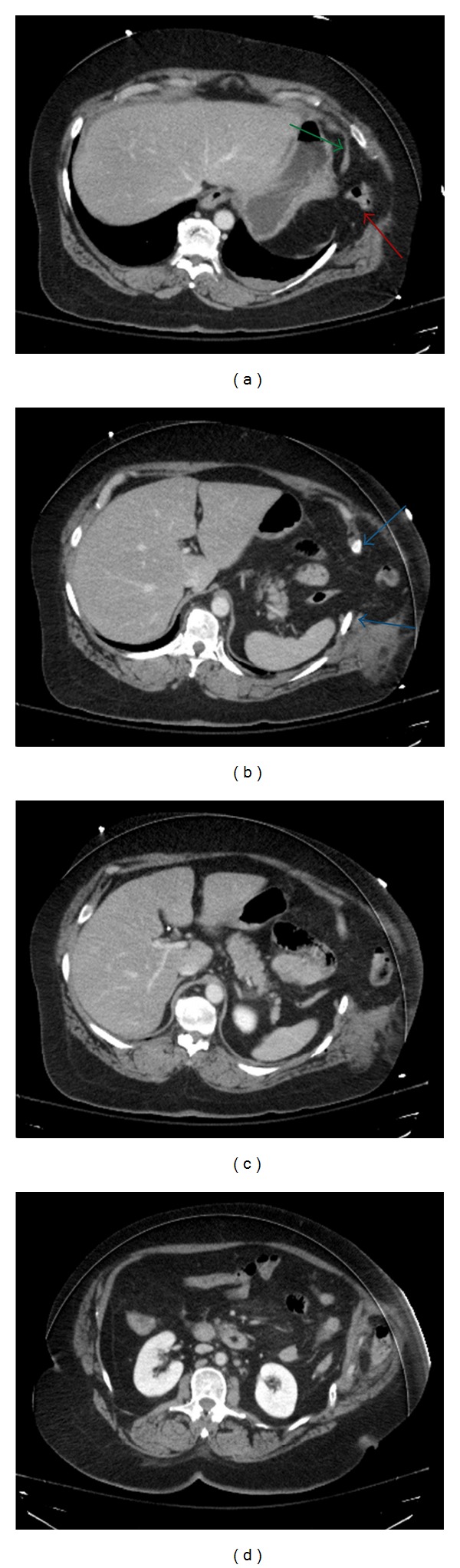
Axial CT images of the abdomen demonstrate herniation of abdominal contents is present including omentum and transverse colon into the chest and through the intercostal defect forming a hernia sac composed of peritoneum (red arrow) (a). There is discontinuity of the diaphragm (green arrows) and widened intercostal space (blue arrows).

**Figure 3:**
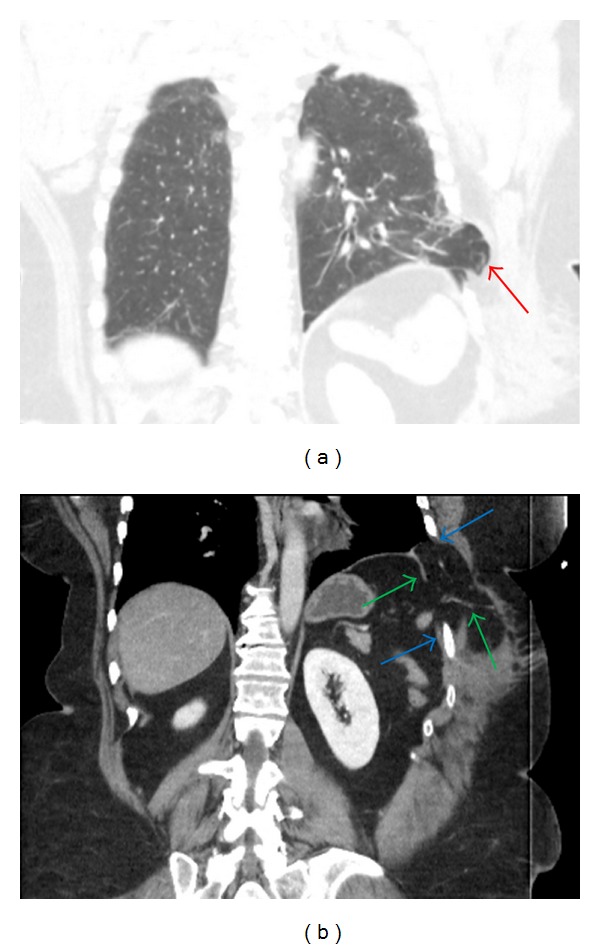
Coronal CT images of the abdomen demonstrate herniation of lung beyond the thoracic cavity (red arrow) (a). There is discontinuity of the diaphragm (green arrows) and widened intercostal space (blue arrows) (b).

Unenhanced CT of the head demonstrated no acute intracranial abnormality. CT of the cervical spine demonstrated several vertebral and transverse processes fractures. A contrast enhanced CT of the chest, abdomen, and pelvis (Figures 1(a)–1(b)) identified fractures of the 7th and 8th ribs with traumatic distraction of the left seventh intercostal space and an associated lateral avulsion of the left hemidiaphragm. This constellation of injuries led to a large, diaphragmatic, and left body wall defect through which the splenic flexure of the colon herniated into the chest (Figures 2(a)–2(d)). Additionally, there was a partial herniation of the left lower lobe (Figures 3(a)–3(b)), proximal left colon, and several small bowel loops at the level of the left body wall (Figures 2(a)–2(d)). There were several additional peritoneal and orthopedic injuries including signs of small bowel mesenteric vascular injury with a large retromesenteric hematoma and a small intraparenchymal contusion of the lateral segment of the left hepatic lobe. Acute fractures of the left L1 transverse process and right femoral shaft below the stem of the femoral head prosthesis were also identified.

The patient was immediately taken to the operating room for exploratory laparotomy of the abdomen and primary repair of the diaphragm and intercostal hernia. Midline abdominal incision from xiphoid to pubis was performed. The abdomen was entered in its midline along the linea alba without difficulty. In the left upper quadrant, the transverse colon and splenic flexure were visualized within the chest. These were brought down into the abdominal cavity with ease. The diaphragmatic defect was noted to be approximately 15 cm in length in the posterolateral aspect. There were also rib fractures that were palpable and the lung tissue could be seen within the chest cavity. The diaphragmatic edges were reapproximated and repaired with continuous suture from the deep portion to the lateral edge encompassing all layers of the diaphragm. A second suture was started at the level of the abdominal wall hernia and brought together the abdominal wall tissue and all layers including where the diaphragm margins approximated the abdominal wall as there was somewhat hockey-shaped defect. A left thoracostomy tube was also placed. 

**Figure 4:**
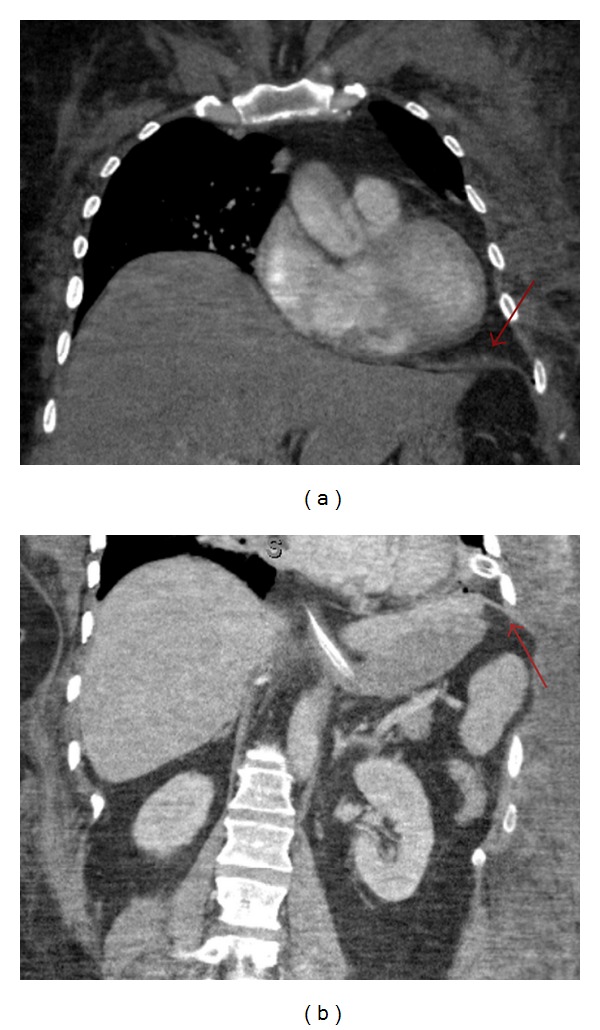
Coronal CT images of the chest and abdomen taken 1 week after surgical repair of the diaphragm (red arrow) show no residual transdiaphragmatic or intercostal herniation.

Within the peritoneal cavity, several adhesions were identified involving the omentum. Additionally, in the left lower quadrant due to her prior surgery, the bowel was densely adherent to abdominal wall and lysis was performed. In the distal jejunum, proximal ileum area, there was a linear mesenteric tear which was not actively bleeding and did not appear to have any vascular compromise to the small bowel in this region. There was no evidence of serosal tears or other bowel injury. Two small 1 cm liver lacerations were identified at the liver edge at approximately segment 5 and segment 3 of the liver and were cauterized. The abdomen was closed without difficulty. Intrathoracic pressures were noted to be stable. On routine postoperative follow-up CT 1 week following surgery (Figures 4(a)–4(b)), there was no residual herniation and the repaired diaphragm was intact.

## 2. Discussion

Extrathoracic lung herniation involves protrusion of pulmonary tissue beyond the thoracic cavity through an abnormal opening in the chest wall [1]. Herniation of the lung through the intercostal muscles is a rare phenomenon, however spontaneous herniation may occur in the presence of local impairment of the thoracic wall with associated increased intrathoracic pressure [2, 3]. This impairment can occur following blunt trauma and be associated with rib fractures resulting in traumatic intercostal hernias [4]. Intercostal herniation may occur following penetrating injury or surgical intervention as well. Hernias are not always associated with rib fractures; they can be either congenital or acquired. Acquired hernias can be spontaneous, posttraumatic, or pathologic as a result of a neoplastic or inflammatory process. Transdiaphragmatic intercostal hernias (TIH) following diaphragmatic rupture or a diaphragmatic defect are particularly rare. Less than 40 cases of TIH have been reported in the literature. [5, 6]. The majority of cases of intercostal herniation, both with or without diaphragmatic injury, occur following blunt trauma. As reported by Unlu et al. in a comprehensive review, advanced age, excessive weight loss, and increased intra-abdominal pressure in addition to trauma are predisposing factors [7].

The pathophysiology of traumatic transdiaphragmatic intercostal herniation specifically involves the forceful tearing of the intercostal muscles as well as the costal attachments of the diaphragm [7]. This is evident in our case as there is both disruption of the diaphragm at the costal margin as well as multiple tears in the intercostal muscles following displaced rib fractures. Blunt or penetrating thoracoabdominal trauma can result in diaphragmatic rupture or injury [1, 3]. Diaphragmatic injury by itself is not uncommon, however in the majority of cases intra-abdominal organs are herniated into the intrathoracic cavity. In cases of penetrating or blunt thoracoabdominal injury, particularly in the lower chest and upper abdomen, a diaphragmatic injury should be suspected and the diaphragm should always be thoroughly examined in order not to miss any small lacerations [8].

Diaphragmatic rupture leads to a weakening in the resistance of the thoracic wall. Furthermore, the integrity of the thoracoabdominal wall is disrupted by the tearing of the intercostal muscles between fractured ribs [5]. Anatomically, the chest wall is weakest anteriorly from the costochondral junction to the sternum, This region is supported by the internal intercostals muscles with a lack of external intercostal muscle support [9]. The most commonly fractured ribs were those between the 8th and 10th with the ninth interspace involved in 59% of the cases [7]. The development of defects in these weakest areas of the chest wall leads to separation of the ribs and the development of a potentially weakened space that is vulnerable and can lead to intercostal herniation of lung tissue or abdominal viscera [10]. Increasing intra-abdominal and intrathoracic positive pressure causes the diaphragmatic defect to progressively enlarge. The transthoracic fascia, pleura, transversalis fascia, and peritoneum form the outer layers of the hernia sac [8].

Transdiaphragmatic intercostal hernias are suggested by the patient's history and physical examination. Clinically, transdiaphragmatic intercostals hernias are visible during suspended respiration on inspiration. Small hernias can be diagnosed only on inspiration. 

Chest radiographs may show herniation of the digestive tract through the chest wall. CT scans are necessary for confirming the diagnosis and for choosing the best curative strategy while determining the extent of associated injuries in the chest, abdomen, and pelvis. [5, 6]. The radiologic findings associated with intercostal trauma include diaphragmatic tears or diaphragm detachments from the costal margins, typically associated with 4 to 30 cm intercostal defects. In addition, abdominal organs may be observed within the abdominal sac [7]. Soft tissue contusion may be present at the site of herniation. Additional associated injuries can include solid organ injury to spleen, liver, pancreas, kidneys, adrenal glands as well as bowel injury, mesenteric contusion, omental contusion, peritoneal bleeding, or retroperitoneal hematoma.

Definitive management of transdiaphragmatic intercostal hernias is achieved through surgical repair. Occasionally spontaneous regression has been observed in small asymptomatic hernias, but large hernias or hernias which have a risk of incarceration must be treated by surgical repair [11, 12] The main cause of mortality however is bleeding from associated injuries [13]. Most reported cases of intercostal herniation have been managed by open surgical repair. Abdominal, thoraco-abdominal, and thoracic approaches have all been utilized for surgical repair [6]. The abdominal approach is often used in traumatic cases, as there are often many associated intra-abdominal injuries. 

In our case, CT had previously showed the presence of mesenteric contusion and retromesenteric hematoma suggesting mesenteric vascular and/or bowel injury. Ultimately treatment requires suturing of the diaphragmatic and the intercostal defects. There is no consensus recommendation regarding the use of prostheses for hernia repair, however the insertion of a nonabsorbable mesh if there is no contamination has been successfully reported in a few instances [6]. Rib fractures are typically not stabilized with reconstruction plates after blunt chest trauma, however this has been successfully performed in isolated cases to reduce blunt chest injury associated morbidity by improving respiratory mechanics and providing pain relief [14]. 

In conclusion, transdiaphragmatic intercostal herniation is a rare clinical entity which can be promptly diagnosed by computed tomography. Diaphragmatic injury should be suspected in all cases of intercostal herniation. Surgical repair of the diaphragm and intercostal defects can result in favorable outcomes.
